# Prevalence and associated factors of active trachoma among children of 1 to 9 years in low-income countries of Africa: a systematic review and meta-analysis

**DOI:** 10.3389/fpubh.2025.1478001

**Published:** 2025-08-11

**Authors:** Leykun Berhanu, Gete Berihun, Belay Desye, Abebe Kassa Geto, Chala Daba

**Affiliations:** ^1^Department of Environmental Health, College of Medicine and Health Sciences, Wollo University, Dessie, Ethiopia; ^2^Department of Environmental Health, College of Medicine and Health Sciences, Debre Markos University, Debre Markos, Ethiopia; ^3^Department of Public Health, College of Health Sciences, Woldia University, Woldia, Ethiopia

**Keywords:** active trachoma, Africa, low-income, children 1 to 9 years, surgery, antibiotics, environmental cleaning

## Abstract

**Background:**

Active trachoma is a form of infectious eye disease caused by *Chlamydia trachomatis*. An estimated 84 million individuals worldwide, primarily children, are affected by active trachoma. Therefore, this systematic review and meta-analysis aimed to determine the pooled prevalence and predictors of active trachoma among children aged 1 to 9 years in low-income countries of Africa.

**Methods:**

Relevant literature was searched from electronic databases. The data was extracted using an Excel sheet and exported to STATA version 17 software. The levels of heterogeneity among studies were assessed using I^2^ and *p*-values. The findings were presented using a table, graph, and forest plot with a 95% confidence interval. A *p*-value of less than 0.05 was considered statistically significant.

**Result:**

Among 2,665 studies searched, 25 were selected for meta-analysis. The pooled prevalence of active trachoma was 21.93% (95% CI; 16.67, 27.20). Presence of fly on child’s face (POR: 2.43: 95% CI; 1.63, 3.24), absence of waste disposal pit (POR: 2.10: 95% CI; 1.36, 2.84), living in rural areas (POR: 0.68: 95% CI; 0.43, 0.93), being female (POR: 1.57: 95% CI; 1.07, 2.07), presence of discharge on the child’s eye (POR: 3.03; 95% CI: 2.20, 3.86), absence of latrine (POR: 1.28: 95% CI; 1.05, 1.50), inadequate knowledge of trachoma (POR: 2.84: 95% CI; 1.69, 3.99), educational status of the child (POR: 0.51: 95% CI; 0.20, 0.81), water consumption (POR: 0.22: 95% CI; 0.04, 0.39), living with animals (POR: 3.35: 95% CI; 2.41, 4.29), latrine utilization (POR: 8.18: 95% CI; 2.16, 14.20), proper latrine utilization (POR: 2.85: 95% CI; 1.84, 3.86), and unclean child face (POR: 0.09: 95% CI; −0.00, 0.19) were the factors significantly associated with the pooled prevalence of active trachoma.

**Conclusion:**

The prevalence of active trachoma among children aged 1 to 9 years is high compared to the World Health Organization trachoma eradication plan. As a result, it is recommended to use latrines and incorporate trachoma awareness into school curricula and community outreach initiatives.

## Introduction

Trachoma is the most common cause of blindness due to a persistent Chlamydial trachomatis of the conjunctiva (1, 2). The World Health Organization’s Global Elimination of Trachoma Alliance has promoted the SAFE (Surgery, Antibiotics, Facial, and Environmental cleaning) strategy to eliminate trachoma as a public health problem (3). This strategy was adopted by WHO in 1996 as a result of recent advances in trachoma control, a standardized surgical procedure for trichiasis, the development of community-based control strategies, new information on trachoma risk factors, and research demonstrating effective azithromycin treatment of active trachoma based on the example given above (4, 5).

Repeated episodes of active trachoma, caused by the bacterium *Chlamydia trachomatis*, can lead to trachomatous trichiasis, and if left untreated, it can result in corneal opacification which leads to vision impairment and even blindness (6). According to the report of World Health Organization 2019, about 27.8 million cases of active trachoma in Africa account for 68.5% of all global cases (7). Further, according to the most recent report of WHO (1), among 114.2 million people exposed to Trachomatous Inflammation-Follicular, 103.3 million (93%) were living in Africa, underscoring the region’s disproportionate vulnerability.

The high prevalence of trachoma among children in many parts of Africa, with rates reaching 60–90%, is alarming and reflects ongoing challenges in achieving the WHO trachoma elimination goal (8). In Ethiopia, the pooled prevalence of active trachoma ranges from 24 to 35.8% (9, 10). Ethiopia, along with Nigeria, Sudan, and Guinea, bears a significant portion of the global burden of active trachoma, collectively accounting for 48.5% of the world’s cases which highlights the pressing need for intensified efforts to combat the disease in these high-burden countries (11, 12).

Trachoma remains a significant public health challenge, particularly in Africa, a comprehensive systematic review and meta-analysis focusing specifically on the prevalence of active trachoma and its associated factors in children aged 1–9 years is notably lacking. Previous studies may have reported a wide range of prevalence estimates for active trachoma which need to be aggregated to produce strong evidence for policymakers. Thus, this systematic review and meta-analysis were conducted to determine the prevalence and associated factors of active trachoma among children of 1 to 9 years in low-income countries of Africa. According to World Bank country classification, low income countries of Africa include, Burkina Faso, Burundi, Central African Republic, Chad, Congo Democratic Republic, Eritrea, Ethiopia, Gambia, Guinea-Bissau, Liberia, Madagascar, Malawi, Mali, Mozambique, Niger, Rwanda, Sierra Leone, Somalia, South Sudan, Sudan, Togo and Uganda (13).

## Materials and methods

### Protocol registration

The protocol for this systematic review and meta-analysis is registered in the International Prospective Register of Systematic Reviews database with registration number CRD42024496870.

### Search strategy

Relevant literature was searched in PubMed, Google, Scholar Google, CINHAL, Hinari, African Journals Online, and Science Direct. Grey literature was also searched. In addition, experts were approached to provide feedback on the search method before initiating database searches. Reference searches were also conducted. The initial search was conducted on advanced PubMed databases using Boolean operators like “OR” and “AND.” The date of searching for papers in each database was noted at the time of searching. The relevant literature was searched on PubMed using the following search terms” ((((((“Ocular trachoma”) OR (“Active trachoma”) OR (“Granular conjunctivitis”) OR (“Infectious trachoma”) OR (“Trachomatous inflammation”) OR (“Acute bacterial conjunctivitis”)))))) AND (((((((“Associated factors”) OR (“Contributing factors”) OR (“Causal factors”) OR (“Influencing factors”) OR (“Determining factors”) OR (“Risk Factors”) OR (“Contributory factors”))))))) AND (((((((((((((((((((((((((((((“Burkina Faso”) OR (Burundi) OR (“Central African Republic”) OR (Chad) OR (“Congo Democratic Republic”) OR (Eritrea) OR (Ethiopia) OR (Gambia) OR (“Guinea-Bissau”) OR (Liberia) OR (Madagascar) OR (Malawi) OR (Mali) OR (Mozambique)) OR (Niger) OR (Rwanda) OR (“Sierra Leone”) OR (Somalia) OR (“South Sudan”) OR (Sudan) OR (Togo) OR (Uganda))))))))))))))))))))))))))))).” By incorporating key terms like active trachoma, predictors, and the name of each low-income country of Africa, searches on additional databases were also conducted. This study followed the updated Preferred Reporting Items for Systematic Review and Meta-analysis (PRISMA) guidelines (Supplementary File 1).

### Inclusion criteria

Study area: Research conducted in low-income countries of Africa.

Types of study included: All observation studies (cross-sectional, case–control, and cohort).

Language: All research written in the English language.

Publication status: All published studies.

Outcome of interest: studies reporting the prevalence of active trachoma among children aged one to nine years.

### Exclusion criteria

Studies focusing on chronic trachoma, and those without full text were excluded. In addition, books, book chapters, annual reports, reviews, letters to editors, and brief communications were also removed. Furthermore, studies that focus on the effectiveness of specific treatments were excluded.

### Outcome assessment

The objective of this research was to assess the pooled prevalence of active trachoma in children aged 1 to 9 years in low-income countries of Africa. The pooled prevalence is estimated by dividing the number of research participants with active trachoma by the actual sample size and multiplying by 100. To identify the factors associated with the pooled prevalence of active trachoma, a systematic review and meta-analysis were conducted.

### Operational definition

Active trachoma was measured as the presence of either trachomatous inflammation follicles and/or Trachomatous inflammation intense (14). Trachoma inflammation follicle is a condition in which 5 or more follicles with a diameter of 0.5 mm or more are present in the central part of the epitaxial conjunctiva (15). Trachomatous inflammation intense refers to inflammatory thickening of the tarsal conjunctiva that obscures more than half of the normal deep tarsal vessels (16).

### Study selection process

**Figure 1:**
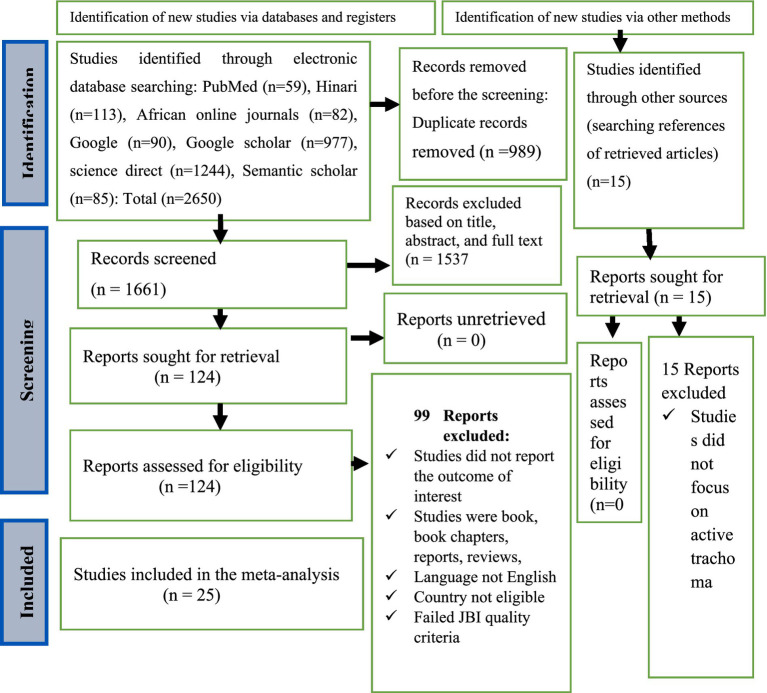
PRISMA flow diagram showing the selection of studies for systematic review and meta-analysis on the prevalence and associated factors of active trachoma among children aged 1 to 9 years in low-income countries of Africa, 2024.

Two researchers, LB and GB independently examined published articles based on their full-text accessibility, abstract, and title. The identified published articles were line listed for further screening. All selection discrepancies and confusions were resolved through consultation with other co-authors. The 2020 PRISMA flow diagram has been used to summarize the study selection procedure (Figure 1).

### Data extraction

After scanning relevant literature in the proposed databases and additional sources, papers were exported to Endnote version 20 software. The amount of duplicate studies deleted from each database was tracked. After deleting all duplicates, the studies were assessed based on titles and abstracts against predetermined study inclusion criteria. A data extraction template was created, which included the author’s name, publication year, sample size, country, type of study design, response rate, active trachoma prevalence, potential risk factors for active trachoma, adjusted odds ratio, and confidence interval for each of the predictor.

### Study quality and bias assessment

The Joanna Briggs Institute quality rating instrument was used to assess the quality of papers for consideration in the review. LB, AKG, and GB independently perform study quality assessments using JBI criteria for cross-sectional and case–control studies. The criteria measured each of the studies out of 100%. Eight criteria make up the critical analysis checklist. Every criterion has a reply option of yes, no, uncertain, and not applicable. After review, studies with a score of 50% and above were identified as having low risk and included in the meta-analysis study (17) (Supplementary File 2). Egger’s and Begg’s tests were utilized to evaluate publication bias objectively. A funnel plot was employed to subjectively evaluate publication bias among the studies incorporated in this study.

### Data analysis and presentation

The data was extracted using an Excel sheet and exported to STATA version 17 software for analysis. Heterogeneity among the selected studies was assessed using I^2^ and *p*-value. Heterogeneity among the included studies was considered high (I^2^ = 99.29, *p* < 0.001). Due to high heterogeneity among the included studies, the random effect meta-regression analysis model was used to measure the pooled prevalence and associated factors of active trachoma. The finding was presented using a table, graph, and forest plot with a 95% confidence interval. A *p*-value of less than 0.05 was considered statistically significant.

## Result

### Search strategy and study selection process

Relevant literature was searched from electronic databases including PubMed, Hinari, African Journal Online, Google, Google Scholar, Science Direct, and Semantic Scholar. In total, 2,665 studies were searched from various electronic databases. Of them, 1,661 studies were selected for further screening and the remaining was excluded because of duplication. Among 1,661 studies, one hundred twenty-four studies were assessed for eligibility and the remaining 1,557 studies were removed because of titles, abstracts, and full-text reviews. Of 124 studies selected for eligibility assessment, 35 were eligible for JBI study quality assessment. Finally, 25 studies were selected for meta-analysis (Figure 1).

### Description of the included studies

Of the 25 studies included in the meta-analysis, 20 of them were published in Ethiopia (7, 14, 15, 18–34), and one study each from Sudan (35), Malawi (36), South Sudan (37), Gambia (38), and Uganda (39). In the 25 studies, the 24,300 study participants were examined for the presence of active trachoma and 8,206 (33.8%) of them were positive. The highest and lowest prevalence of active trachoma was 64.5% (37) and 1.3% (39), respectively (see Table 1).

**Table 1:** Characteristics of the included studies reporting the prevalence and associated factors of active trachoma among children aged 1–9 years in low-income countries of Africa, 2024.

Author (year)	Country	Study design	Diseased	Prevalence of active trachoma	Sample size	Response rate (%)	JBI score (100%)
Abdilwohab and Abebo (2020) (14)	Ethiopia	Cross-sectional	148	17.8	831	91.6	62.5
Adane et al. (2023) (15)	Ethiopia	Case–control	242	33.3	726	98.4	70
Alambo et al. (2020) (18)	Ethiopia	Cross-sectional	222	37.9	586	100	75
Alemayehu et al. (2015) (7)	Ethiopia	Cross-sectional	105	15.6	671	96.5	62.5
Alkhidir et al. (2018) (35)	Sudan	Cross-sectional	99	11.0	900	100	75
Asmare et al. (2023) (19)	Ethiopia	Cross-sectional	191	35.4	540	92.3	75
Asres et al. (2016) (20)	Ethiopia	Cross-sectional	71	12.1	586	98.1	75
Ayelgn et al. (2021) (21)	Ethiopia	Cross-sectional	89	11.8	752	94.9	75
Belsti et al. (2021) (22)	Ethiopia	Cross-sectional	132	21.6	610	98.39	100
Delelegn et al. (2021) (23)	Ethiopia	Cross-sectional	123	17.5	701	94	62.5
Kalua et al. (2010) (36)	Malawi	Cross-sectional	435	17.9	2,430	100	50
Kedir et al. (2021) (24)	Ethiopia	Cross-sectional	165	29.4	561	95.2	62.5
Mekonnen et al. (2022) (25)	Ethiopia	Cross-sectional	39	21.9	178	100	62.5
Mengistu et al. (2016) (26)	Ethiopia	Cross-sectional	224	36.7	611	98.87	50
Mohamed et al. (2019) (27)	Ethiopia	Cross-sectional	35	4.3	823	97.2	50
Ngondi et al. (2007) (37)	South Sudan	Cross-sectional	4,785	64.5	7,418	100	50
Nigusie et al. (2015) (28)	Ethiopia	Cross-sectional	143	23.1	618	100	75
Quicke et al. (2013) (38)	Gambia	Cross-sectional	25	3.8	652	100	50
Reda et al. (2020) (29)	Ethiopia	Cross-sectional	108	21.5	502	100	75
Tadesse et al. (2017) (30)	Ethiopia	Cross-sectional	293	21.6	1,358	100	75
Tuke et al. (2023) (31)	Ethiopia	Cross-sectional	157	29.2	538	99.6	75
W/Hana et al. (2023) (32)	Ethiopia	Cross-sectional	77	17.8	433	96.2	75
Woldekidan et al. (2019) (33)	Ethiopia	Cross-sectional	87	15.2	574	97.4	87.5
Yeshitila et al. (2022) (34)	Ethiopia	Cross-sectional	205	27.0	760	100	87.5
Ndikuno et al. (2022) (39)	Uganda	Cross-sectional	6	1.3	472	59.1	50

### Meta-analysis

#### Pooled prevalence of active trachoma

**Figure 2:**
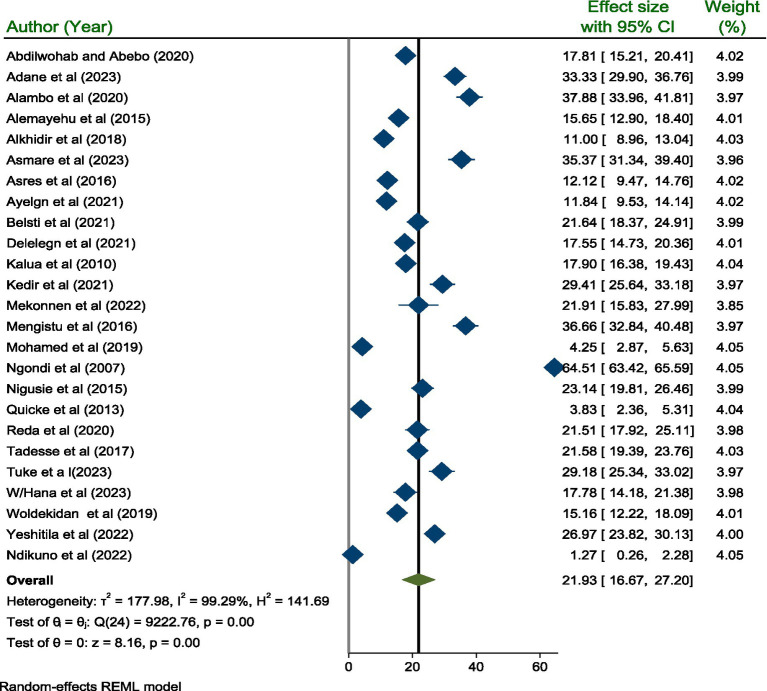
A forest plot showing the pooled prevalence of active trachoma among children 1–9 years old in low-income countries of Africa, 2024.

The pooled prevalence of active trachoma among children aged 1 to 9 years was 21.93% (95% CI; 16.67, 27.20). Due to the high level (I^2^ = 99.29%, *p* < 0.001) of heterogeneity among the included studies, a random effect model was used to estimate the pooled prevalence of active trachoma (Figure 2).

#### Publication bias assessment

**Figure 3:**
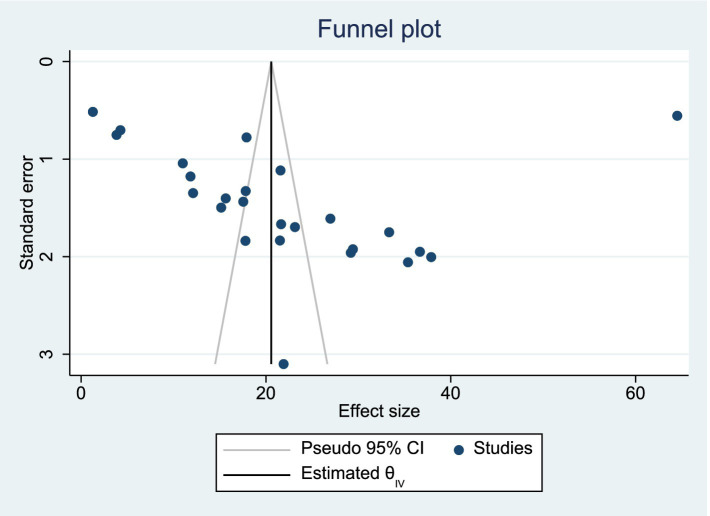
Funnel plot showing absence of publication bias among studies included in the systematic review and meta-analysis.

We used Egger’s test to assess for potential publication bias. This review showed no publication bias (*p* = 0.906) (see Figure 3).

#### Sensitivity analysis

**Figure 4:**
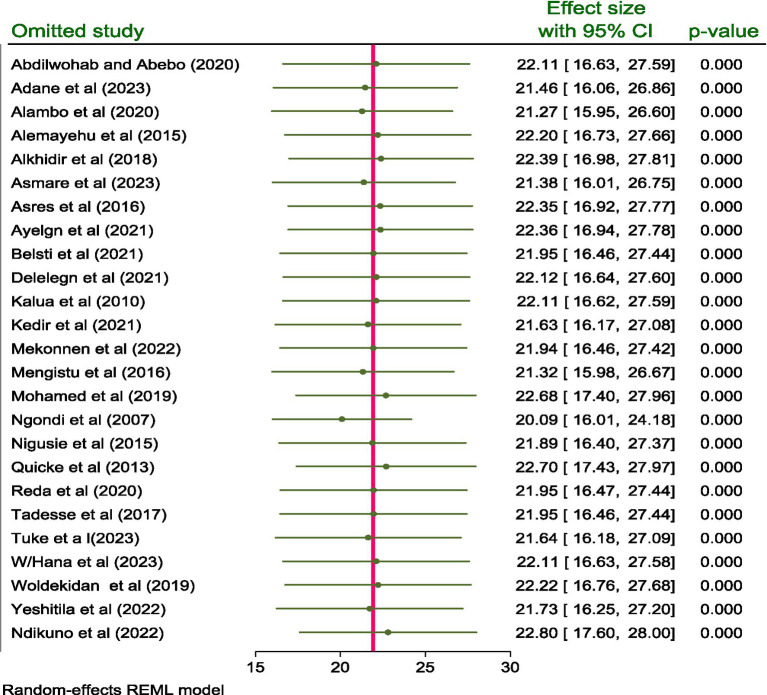
Sensitivity analysis for the prevalence and associated factors of active trachoma among children aged 1 to 9 years in low-income countries of Africa, 2024.

The impact of a single study effect on the pooled prevalence of active trachoma was checked using sensitivity analysis. As indicated below in the figure, when the first study (14) was omitted from the model, the pooled prevalence of active trachoma was 22.11% (95% CI; 16.63, 27.59) and when the 2nd study (15) was removed from the model, the pooled prevalence of active trachoma was reported to be 21.46% (95% CI; 16.06, 26.86). This prevailed that the pooled prevalence of active trachoma among children aged 1 to 9 years in low-income countries of Africa was not affected by a single study included in the review (*p* < 0.001) (Figure 4).

#### Subgroup analysis

**Figure 5:**
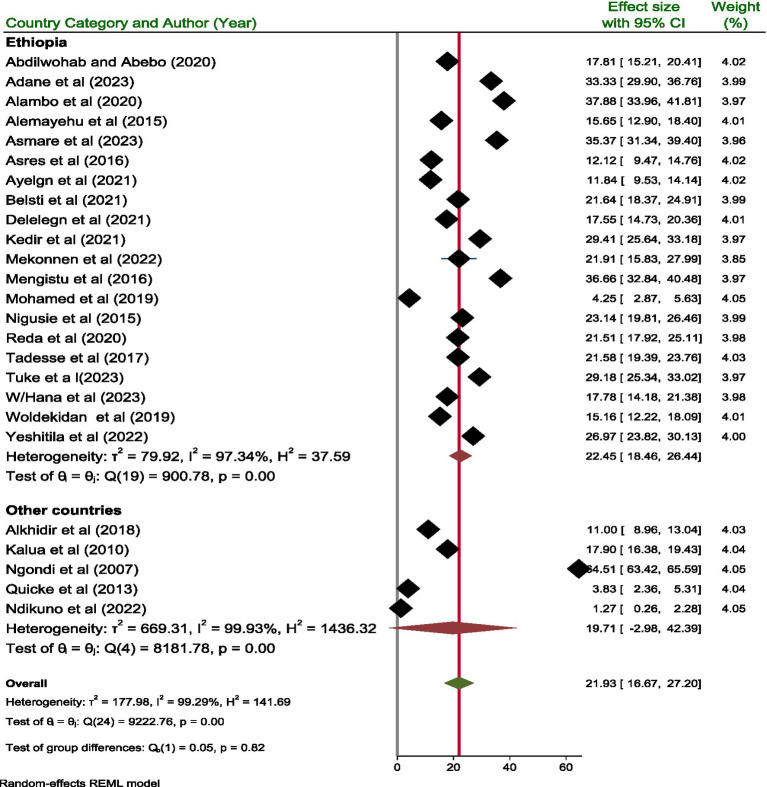
Subgroup analysis based on country classification.

A subgroup analysis was performed based on the publication year, country, and categories of sample size. Based on the country, the highest and lowest pooled prevalence of active trachoma was reported in Ethiopia and other countries with a prevalence of 22.45% (95% CI; 18.46, 26.44) and 19.71% (95% CI; −2.98, 42.39), respectively. In addition, the pooled prevalence of active trachoma reported from studies done in Ethiopia and other countries showed significant difference (*p* = 0.001) (Figure 5).

**Figure 6:**
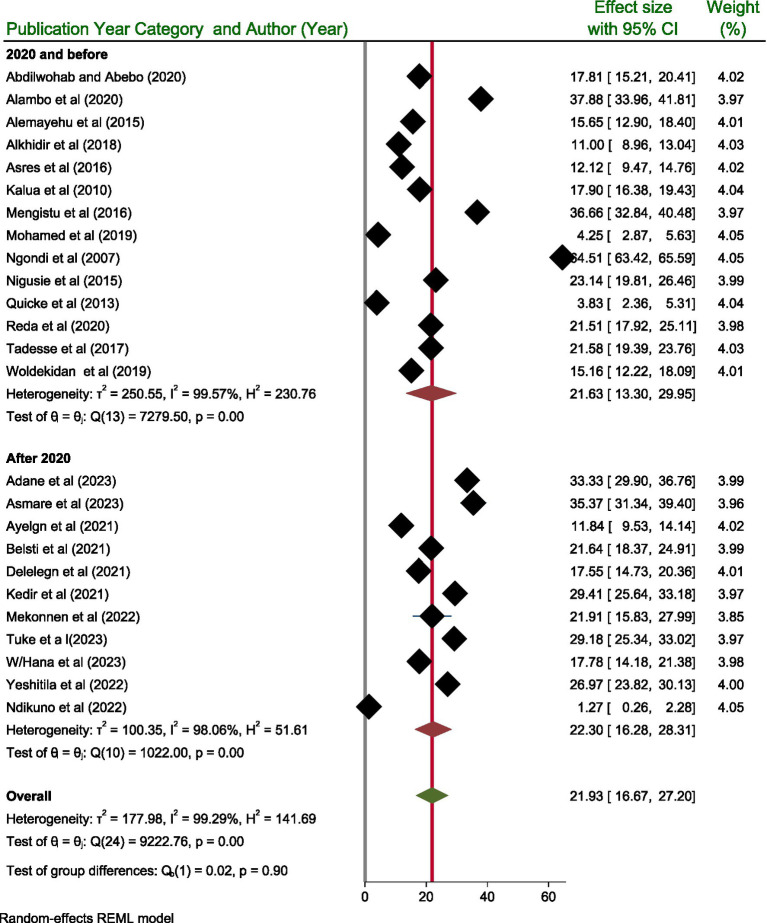
Subgroup analysis result based on publication year category.

Taking into account the publication year category, the highest prevalence of active trachoma was reported after 2020, with a prevalence of 22.30% (95% CI: 16.28, 28.31) among children aged 1–9 years, as opposed to 21.63% (95% CI: 13.30, 29.95) among those study published/done before 2020. However, the test of group difference showed that the pooled prevalence active trachoma did not show significant difference (*p* = 0.90) (Figure 6).

**Figure 7:**
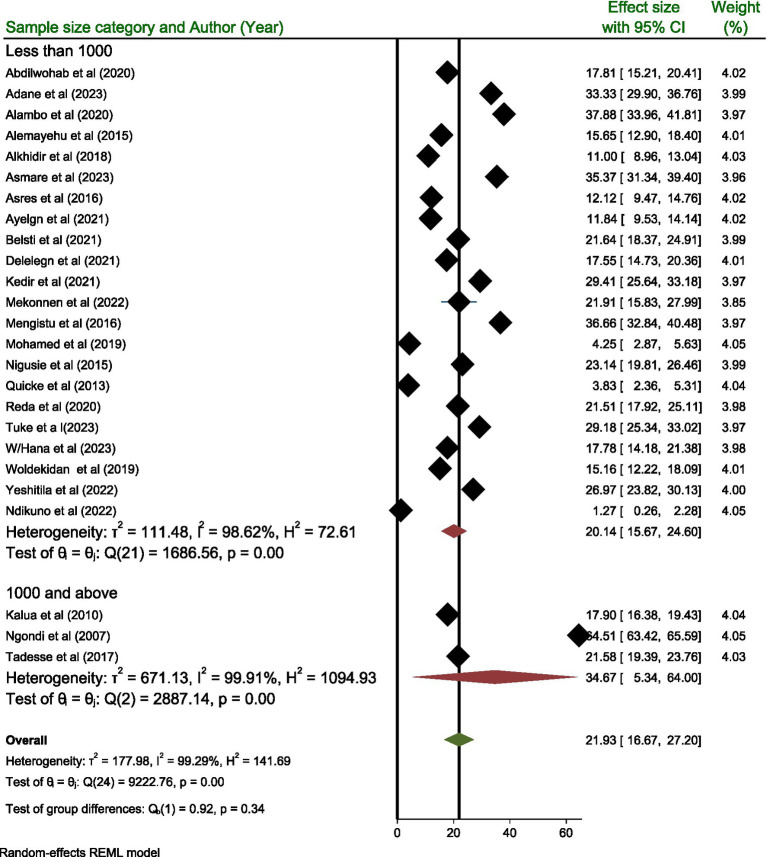
Subgroup analysis result based on sample size category.

Subgroup analysis performed based on the sample size category revealed that the highest prevalence of active trachoma was reported in those categories containing a sample size of 1,000 and above with a prevalence rate of 34.67% (95% CI; 5.34, 64.00) compared to those categories containing a sample size below 1,000 where the prevalence is reported to be 20.14% (95% CI; 15.67, 24.60) (Figure 7).

#### Univariate-meta-regression analysis

The heterogeneity assessment indicated a high level of heterogeneity (I^2^ = 99.29%, *p* < 0.001) among the included studies. The country category, sample size, and publication year were used in a meta-regression analysis to investigate the source of heterogeneity. The analysis revealed that there was no statistically significant (*p* > 0.05) cause of heterogeneity in the included studies (Table 2).

**Table 2:** Univariate meta-regression analysis.

Variables	Coefficient	Std. error	95% CI	*p*-value
Publication year	3.484483	5.461151	−7.22,14.19	0.523
Country	9.116124	7.057058	−4.72, 22.95	0.196
Sample size	21.09391	8.921391	−2.99,24.13	0.018

#### Factors associated with active trachoma

Among 32 potential predictors of active trachoma extracted for analysis, 13 predictors had a statistically significant association with the pooled prevalence of active trachoma. Those factors includes; presence of flies on children’s faces (yes), utilization of waste disposal pit (no), place of residence (rural), sex of the selected child (female), presence of discharge on children’s eye (yes), presence of latrine (no), knowledge of trachoma (inadequate), educational status of the child (enrolled), water consumption (20-40 L), living with animal (yes), all family members use latrine (no), proper latrine utilization (no), and unclean child face (no).

The association between the presence of flies on a child’s face and the pooled prevalence of active trachoma was also investigated using four studies (23, 25, 26, 34). The analysis revealed that children with flies on their faces were 2.43 times more likely to develop active trachoma than children who did not have flies on their faces (POR: 2.43; 95% CI; 1.63, 3.24). The association between the utilization of waste disposal pits and the pooled prevalence of active trachoma was assessed using four studies (19, 20, 24, 33). The analysis revealed that households lacking waste disposal pits had 2.10 times greater likelihood of developing active trachoma than those who utilized them (POR; 2.10: 95% CI; 1.36, 2.84).

The association between place of residence and pooled prevalence of active trachoma among children was also assessed using three studies (19, 22, 24). The finding showed that households who lived in rural areas were 32% less likely to develop active trachoma than those who lived in urban areas (POR: 0.68; 95% CI; 0.43, 0.93). The presence of a statistically significant association between the sex of the selected child and the pooled prevalence of active trachoma was assessed by including four studies (20, 23, 24, 39). The finding indicated that female children were 1.57 more likely to develop active trachoma than male children (POR: 1.57:95% CI; 1.07, 2.07).

The association between the pooled prevalence of active trachoma and the presence of discharge in the child’s eye was also studied using seven studies (7, 19, 23, 30, 32, 38, 39). The finding indicated that those children who had discharge on their eyes were 3.03 more likely to develop active trachoma as compared to those who did not have discharge on their eyes (POR: 3.03; 95% CI: 2.20, 3.86). The association between the pooled prevalence of active trachoma and the presence of latrine was also studied using seven studies (18, 20, 24, 27, 29, 31, 34). The findings indicated that households without a latrine facility were 1.28 times more likely to develop active trachoma compared to those with a latrine facility (POR: 1.28; 95% CI: 1.05, 1.50). The association between the pooled prevalence of active trachoma and knowledge of trachoma was checked using two studies (26, 29). The report showed that households with inadequate knowledge about trachoma were 2.84 times more likely to develop active trachoma when compared with those who had adequate knowledge about trachoma (POR; 2.84; 5% CI; 1.69, 3.99).

In addition, the association between the educational status of the children and the pooled prevalence of active trachoma was also assessed using two studies (18, 20). The assessment revealed that children who enrolled in school were 49% less likely to develop active trachoma than those not enrolled (POR: 0.51; 95% CI; 0.20, 0.81). The association between the pooled prevalence of active trachoma and consuming 20 to 40 liters of water per day was assessed by including two studies (18, 22). The assessment indicated that households who consumed 20 to 40 liters of water per day were 78% less likely to develop active trachoma as compared to those who consumed less than 20 liters of water per day (POR:0.22:95% CI 0.04, 0.39).

The association between the pooled prevalence of active trachoma and living with animals was also studied using two studies (31, 34). The finding indicated that those households living with animals were 3.35 times more likely to develop active trachoma than those who did not live with animals (POR: 3.35; 95% CI; 2.41, 4.29). The association between the pooled prevalence of active trachoma and latrine utilization of the family members has also been assessed in this review using three studies (28, 32, 33). The results showed that all households lacking latrine use were 8.18 times more prone to experiencing active trachoma compared to those that utilized latrine facilities (POR: 8.18; 95% CI; 2.16, 14.20). The association between proper latrine utilization and the pooled prevalence of active trachoma were studied using three studies (14, 19, 21). The association between the pooled prevalence of active trachoma and the cleanliness of a child’s face was also studied by including nine studies (7, 14, 20, 25, 26, 29, 34, 38, 39). The report indicated that children who had clean faces were 99.91% less likely to develop active trachoma as compared to those who had unclean faces (POR: 0.09; 95% CI; −0.00, 0.19) (see Table 3).

**Table 3:** Factors associated with the pooled prevalence of active trachoma among children aged 1 to 9 years in low-income countries of Africa, 2024.

List of variables		Number of participants	No of studies	Pooled odds ratio (95% CI)	Heterogeneity	
					I^2^	*p*-value
Age of children (1–5 years)		1,238	2	2.12 (0.87,3.37)	0.0	0.462
Nasal discharge (yes)		3,373	4	1.27 (0.85,1.68)	31.9	0.221
Presence of fly on child’s face (yes)		2,250	4	2.43 (1.63, 3.24)*	44.4	0.145
Utilization of waste disposal pit (no)		2,261	4	2.10 (1.36,2.84)*	0.0	0.462
Place of residence (rural)		1711	3	0.68 (0.43,0.93)*	0.0	0.731
Sex of the selected child (female)		2,320	4	1.57 (1.07,2.07)*	0.0	0.969
Children use soap when washing his/her face (do not use soap)		3,460	7	0.92 (0.64,1.21)	57.6	0.028
Family size (>5)		2,767	4	1.97 (1.00, 2.94)	6.4	0.361
Selected child age (below five years)		974	2	4.99(−1.07,11.05)	0.0	0.944
Educational status of the mother/caregiver (cannot read and write)		1853	3	1.60 (0.95,2.25)	0.0	0.589
Presence of discharge on the child’s eye (yes)		4,827	7	3.03 (2.20,3.86)*	42.1	0.110
Presence of latrine (no)		4,356	7	1.28 (1.05,1.50)*	41.7	0.113
Domestically produced waste disposal (simple disposal)		1,698	3	1.78 (0.59,2.97)	54.8	0.110
Knowledge of trachoma (inadequate)		113	2	2.84 (1.69,3.99)*	32.1	0.225
Educational status of the child (enrolled)		1,172	2	0.51 (0.20,0.81)*	79.2	0.028
Wealth index of the family	Poor	1,287	2	0.96 (0.52,1.39)	94.9	<0.001
	Medium	1,287	2	0.83 (0.36,1.30)	0.0	0.709
Time to obtain water (> 30 min)		1,417	2	0.60 (0.07,1.14)	85.3	0.009
Water consumption (20–40 L)		1,196	2	0.22 (0.04,0.39)*	25.4	0.247
Water consumption (40–60 L per HH)		1,362	2	0.61 (0.10,1.12)	71.5	0.061
Average daily water consumption (<20 L/c/d)		1,336	2	0.68 (0.02,1.34)	57.2	0.126
Distance of water source (> 30 min)		3,887	5	2.11 (1.00,3.21)	56.3	0.058
Face washing habit (none)		1,236	3	3.33 (0.67, 5.99)	0.0	0.598
Frequency of face washing (only occasionally)		1,298	2	1.12 (0.87,1.37)	0.0	0.666
Face washing habit (once a day)		4,131	7	0.96 (0.58,1.35)	47.0	0.079
Knowledge of mothers/caregivers about TF (poor)		1,312	2	2.75(−0.04,5.54)	0.0	0.474
Source of water (protected spring)		1,392	2	1.55 (0.78,2.33)	54.1	0.140
Cattle living with animals (yes)		1,298	2	3.35 (2.41,4.29)*	80.9	0.022
Mechanism of disposing of dry waste (improper)		2,201	3	1.99 (1.00,2.97)	77.0	0.013
All family members use latrine (no)		1,625	3	8.18 (2.16,14.20)*	0.0	0.871
Proper latrine utilization (no)		2,123	3	2.85 (1.84,3.86)*	15.7	0.305
Unclean child face (no)		5,263	9	0.09(−0.00,0.19)*	85.8	<0.001

*Indicates the presence of a statistically significant association.

## Discussion

Trachoma remains a leading cause of preventable blindness in Africa. The problem worsens if not detected and intervened early in children (10). This study aimed to determine the pooled prevalence of active trachoma and associated factors among children aged 1 to 9 years in low-income countries of Africa. The pooled prevalence of active trachoma among children aged 1 to 9 years in the present study was 21.93% (95% CI; 16.67, 27.20). This figure is lower compared to other findings including; Ethiopia 26.9, 52.4, and 22% (10, 40, 41), Nepal 23.6% (42), and Eastern Colombia 24.9% (43). On the other hand, a lower prevalence was reported for example in Ethiopia at 4.1% (44), Senegal at 8.3% (45), Rio Negro Basin at 11.1% (46), Tanzania at 6.7% (47), and 18.4% (48), and Gambia at 10.4% (49). The low prevalence of active trachoma indicates significant public health improvements, such as better hygiene, sanitation, and access to clean water, leading to a reduced healthcare burden and economic benefits (10). The current results show that the prevalence of active trachoma is four times greater than the eradication target set by WHO, necessitating prompt intervention (49).

The pooled prevalence of active trachoma for studies published after 2020 was calculated to be 22.30% (95% CI; 16.28–28.31). This figure indicates a notable increase in the prevalence of active trachoma in more recent studies than those published before 2020. This percentage indicates a significant increase in the prevalence of active trachoma in more recent research compared to the studies before 2020. This upward trend suggests that active trachoma may be more common in the populations studied, highlighting potential public health concerns. Understanding the significance of this rise is essential, as it may indicate alterations in the dynamics of disease transmission, differences in environmental conditions, or changes in healthcare accessibility and interventions. According to WHO, 61 million people require treatment through the SAFE strategy (49).

Among the countries studied, Ethiopia stands out with the highest recorded prevalence of active trachoma, reported at 22.45% (95% CI; 18.46, 26.44). This rate surpasses the average prevalence of 16.84% observed in other countries included in the analysis. The elevated prevalence in Ethiopia may be attributed to environmental conditions, healthcare access, and socio-economic challenges that hinder effective disease control measures (50, 51).

The present study indicated that households who did not use latrines were 8.18 times more likely to develop active trachoma than those who used latrines (POR: 8.18; 95% CI; 2.16, 14.20). This finding is consistent with previous report (52, 53). A significant association between utilizing latrines and the occurrence of active trachoma indicates that poor sanitation practices may increase the risk of this eye infection. This finding emphasizes the need for public health interventions to promote latrine use, improve sanitation facilities, and educate communities. It also suggests that addressing cultural and economic barriers to latrine usage could be crucial in reducing trachoma incidence, thereby informing health policies and funding priorities aimed at enhancing sanitation as part of broader disease prevention strategies (54, 55).

This systematic review and meta-analysis study revealed that the unclean face of children was significantly associated with the pooled prevalence of active trachoma (POR: 0.09; 95% CI; −0.00, 0.19). This finding is supported by a systematic review and meta-analysis study in Ethiopia (10). Another systematic review and meta-analysis also confirmed that having a clean face was significantly associated with reduced odds of active trachoma (55). Children’s unclean faces can harbor the bacteria that cause trachoma, making hygiene crucial for prevention. Dirty faces, especially with nasal and eye secretions, facilitate the spread of the bacteria to others through direct contact or flies. This ongoing transmission, particularly among young children, complicates efforts to control trachoma and contributes to its prevalence in the community (56).

The present study indicated that the absence of a latrine significantly contributed to the increased pooled prevalence of active trachoma (POR: 1.28; 95% CI; 1.05, 1.50). This is consistent with the systematic review and meta-analysis done in Ethiopia (10) and the global review (57). The finding is also supported by a study in the Gazegibela district of Wagehemra Zone, Ethiopia (16). Without latrines, human waste contaminates soil and water, increasing the spread of trachoma-causing bacteria and making proper hygiene practices difficult. This fosters close contact with infected individuals or contaminated surfaces, while open defecation breeds flies that can transfer bacteria to healthy people (55).

This study indicated that ocular discharge was significantly associated with the pooled prevalence of active trachoma (POR: 3.03; 95% CI: 2.20, 3.86). This finding is supported by the systematic review and meta-analysis study (55). The finding that ocular discharge is significantly associated with the pooled prevalence of active trachoma indicates that individuals with this symptom are at a higher risk for the disease. This underscores the importance of monitoring ocular discharge as a potential early indicator for active trachoma, enabling timely diagnosis and intervention. Public health strategies can be tailored to educate communities about the risks linked to ocular discharge and promote better hygiene practices to reduce transmission. Additionally, health resources may be more effectively allocated to areas with higher instances of ocular discharge to prevent outbreaks. Overall, this finding highlights the need for vigilant health monitoring and targeted interventions to mitigate the risk of active trachoma (58, 59).

In this study, households living with animal had higher odds of developing active trachoma as compared to those who did not live with animal (POR:3.3.5:95% CI; 2.41, 4.29). The presence of statistical association between cattle ownership and active trachoma has also been reported by different studies in Africa (17, 60). In arid environments, cattle droppings provide an ideal habitat for breeding flies, which has been linked to the occurrence of trachoma. This association arises from the understanding that flies can act as vectors for the bacteria responsible for the infection. While some studies have suggested that cattle and fly populations may independently predict trachoma incidence, the prevailing explanation emphasizes the role of cattle in enhancing fly breeding conditions, thereby increasing the risk of disease transmission (61).

The strength of this study is its comprehensive data analysis, which enhances the reliability of prevalence estimates. The findings directly contribute to the implementation and evaluation of the WHO’s SAFE strategy for trachoma elimination. The review provides evidence-based recommendations for policymakers, informs targeted interventions, and reveals research gaps that can guide future studies, thereby strengthening efforts to combat trachoma. The insights could help tailor the SAFE interventions to the specific needs of children aged 1 to 9 years in low-income countries of Africa.

However, most of the studies in this review are cross-sectional, which may restrict the capacity to deduce causal links between the exposures and the desired outcome. The lack of longitudinal research might make it more difficult to evaluate the long-term patterns, trajectories, and dynamic character of the result of interest. Longitudinal data may offer additional information about the evolution, course, and possible risk factors. Furthermore, as longitudinal studies can shed light on the dynamic character of the result and support the establishment of causal linkages, it is advised to incorporate them into future reviews.

## Conclusion

The prevalence of active trachoma is higher compared to the trachoma elimination goal of the World Health Organization. Types, presence, and utilization of latrine and waste disposal pit, place of residence, sex of the selected child, presence of fly on child’s face, availability of latrine, knowledge of trachoma, presence of discharge on the child’s eye, water consumption, living with animals, and unclean child face were the factors significantly associated with the pooled prevalence of active trachoma among children aged 1 to 9 years in low-income countries of Africa.

Hence, it is recommended to promote the construction and use of household latrines, educate mothers and children about the causes, transmission, and prevention of trachoma, and integrate trachoma education into school curricula and community outreach initiatives. Additionally, encouraging frequent face-washing and personal hygiene practices through educational campaigns, and stressing the value of keeping children’s faces clean all contribute to lowering the prevalence of active trachoma in low-income countries of Africa.

## Data Availability

The original contributions presented in the study are included in the article/Supplementary material, further inquiries can be directed to the corresponding author.
