# Supplementary material for: Prevalence and associated factors of active trachoma among children of 1 to 9 years in low-income countries of Africa: a systematic review and meta-analysis
Source: Front Public Health. 2025 Aug 11;13:1478001. doi: 10.3389/fpubh.2025.1478001 (PMC12375597; doi:10.3389/fpubh.2025.1478001)
Supplement: Supplementary file 2 [file Table_2.docx]

**JBI Quality Assessment Criteria**

| Included articles | Criteria | | | | | | | | | |
| --- | --- | --- | --- | --- | --- | --- | --- | --- | --- | --- |
|  | Clear eligibility criteria | Description of study subject and study setting | Valid & reliable method to measure the exposure | Standard criteria used for measurement of the condition | Identification of confounding factors | Development of strategies to deal with confounding factors | Valid and reliable method to measure outcomes | Appropriate statistical analysis | Total score out of (n=8) | Quality Score (100%) |
| Abdilwohab and Abebo, 2020 | YES | YES | YES | NO | NO | YES | NO | YES | 5 | 62.5 |
| Adamu and Fereji 2014 | NO | YES | YES | YES | NO | NO | NO | NO | 3 | 37.5 |
| Ahmed et al. 2016 | NO | NO | YES | NO | NO | YES | NO | NO | 2 | 25 |
| Alambo et al, 2018 | YES | YES | NO | YES | NO | YES | YES | YES | 6 | 75 |
| Alemayehu et al, 2015 | YES | YES | NO | NO | NO | YES | YES | YES | 5 | 62.5 |
| Alkhidir et al, 2018 | NO | YES | YES | YES | NO | YES | YES | YES | 6 | 75 |
| Anteneh et al, 2016 | NO | NO | NO | YES | NO | NO | YES | YES | 3 | 37.5 |
| Asmare et al, 2023 | YES | YES | YES | YES | NO | NO | YES | YES | 6 | 75 |
| Asres et al, 2016 | NO | YES | YES | YES | NO | YES | YES | YES | 6 | 75 |
| Ayelgn et al. 2021 | YES | YES | NO | YES | NO | YES | YES | YES | 6 | 75 |
| Belsti et al, 2021 | YES | YES | YES | YES | YES | YES | YES | YES | 8 | 100 |
| Cumberland et al, 2005 | NO | YES | NO | YES | NO | NO | YES | NO | 3 | 37.5 |
| Delelegn et al, 2021 | YES | YES | YES | NO | NO | YES | NO | YES | 5 | 62.5 |
| Kalua et al, 2010 | NO | YES | YES | YES | NO | NO | YES | NO | 4 | 50 |
| Karimurio et al, 2006 | NO | YES | NO | YES | NO | NO | NO | NO | 2 | 25 |
| Kedir et al, 2021 | NO | YES | YES | YES | NO | YES | NO | YES | 5 | 62.5 |
| Mekonnen et al, 2022 | YES | YES | YES | NO | NO | NO | NO | YES | 5 | 62.5 |
| Mengistu et al, 2016 | NO | YES | NO | YES | NO | NO | YES | YES | 4 | 50 |
| Kassaw et al, 2020 | YES | NO | NO | YES | NO | NO | NO | YES | 3 | 37.5 |
| Mohamed et al, 2019 | NO | YES | NO | YES | NO | NO | YES | YES | 4 | 50 |
| Nasieku et al, 2017 | NO | YES | NO | YES | NO | NO | YES | NO | 3 | 37.5 |
| Ndikuno et al, 2022 | YES | YES | NO | NO | NO | YES | NO | YES | 4 | 50 |
| Ngondi et al, 2007 | YES | YES | YES | NO | NO | YES | NO | NO | 4 | 50 |
| Nigusie et al, 2015 | YES | YES | NO | YES | NO | YES | YES | YES | 6 | 75 |
| Nyamwaro et al, 2011 | YES | NO | YES | NO | NO | NO | NO | NO | 2 | 25 |
| Quicke et al, 2013 | NO | YES | NO | YES | NO | YES | NO | YES | 4 | 50 |
| Qureshi et al, 2010 | YES | NO | NO | YES | NO | NO | NO | YES | 3 | 37.5 |
| Reda et al, 2020 | YES | YES | YES | NO | NO | YES | YES | YES | 6 | 75 |
| Tadesse et al, 2017 | YES | YES | NO | YES | NO | YES | YES | YES | 6 | 75 |
| Edwards et al, 2012 | NO | YES | NO | YES | NO | NO | NO | NO | 2 | 25 |
| Tuke et al, 2023 | YES | YES | YES | NO | NO | YES | YES | YES | 6 | 75 |
| W/Hana et al, 2023 | YES | YES | NO | YES | NO | YES | YES | YES | 6 | 75 |
| Woldekidan et al, 2019 | YES | YES | YES | YES | NO | YES | YES | YES | 7 | 87.5 |
| Yeshitila et al, 2022 | YES | YES | YES | YES | NO | YES | YES | YES | 7 | 87.5 |

**JBI Quality Assessment Criteria for case control study**

| Included articles | Criteria | | | | | | | | | | | |
| --- | --- | --- | --- | --- | --- | --- | --- | --- | --- | --- | --- | --- |
|  | Were the groups comparable other than the presence of disease in cases or the absence of disease in controls? | Were cases and controls matched appropriately? | Were the same criteria used for identification of cases and controls? | Was exposure measured in a standard, valid and reliable way? | Was exposure measured in the same way for cases and controls? | Were confounding factors identified? | Were strategies to deal with confounding factors stated? | Were outcomes assessed in a standard, valid and reliable way for cases and controls? | Was the exposure period of interest long enough to be meaningful? | Was appropriate statistical analysis used? | Total score out of (n=10) | Quality Score (100%) |
| Adane et al, 2023 | YES | YES | YES | YES | YES | NO | NO | YES | NO | YES | 7 | 70 |
